# Graphene in 3D Bioprinting

**DOI:** 10.3390/jfb15040082

**Published:** 2024-03-25

**Authors:** Rahul Patil, Stella Alimperti

**Affiliations:** 1 Department of Biochemistry and Molecular & Cellular Biology, Georgetown University, Washington, DC 20057, USA; rp1144@georgetown.edu; 2 Center for Biological and Biomedical Engineering, Georgetown University, Washington, DC 20057, USA

**Keywords:** 3D bioprinting, graphene, regeneration, cardiovascular, bone, skin, neurons

## Abstract

Three-dimensional (3D) bioprinting is a fast prototyping fabrication approach that allows the development of new implants for tissue restoration. Although various materials have been utilized for this process, they lack mechanical, electrical, chemical, and biological properties. To overcome those limitations, graphene-based materials demonstrate unique mechanical and electrical properties, morphology, and impermeability, making them excellent candidates for 3D bioprinting. This review summarizes the latest developments in graphene-based materials in 3D printing and their application in tissue engineering and regenerative medicine. Over the years, different 3D printing approaches have utilized graphene-based materials, such as graphene, graphene oxide (GO), reduced GO (rGO), and functional GO (fGO). This process involves controlling multiple factors, such as graphene dispersion, viscosity, and post-curing, which impact the properties of the 3D-printed graphene-based constructs. To this end, those materials combined with 3D printing approaches have demonstrated prominent regeneration potential for bone, neural, cardiac, and skin tissues. Overall, graphene in 3D bioprinting may pave the way for new regenerative strategies with translational implications in orthopedics, neurology, and cardiovascular areas.

## 1. Introduction

Three-dimensional (3D) bioprinting is a novel rapid fabrication approach, allowing the development of new implants and transplants for organ restoration and regeneration. Traditional methods for developing 3D tissue-engineered scaffolds involve solvent casting [1], freeze-drying [2], and salt leaching [3,4]. Those methods have demonstrated limitations in generating multifunctional and multi-material scaffolds with controlled geometry, pore network, and size [5,6]. However, recent advances in 3D bioprinting offer the ability to engineer next-generation 3D tissue constructs and implants in a quick and cost-effective manner [7,8]. In general, 3D printing in medicine has been involved in engineering probes and tools for medical testing [9], orthoses and prostheses [10], anatomical models [11], and medical instruments for diagnosis and surgery [12,13]. Recently, it has been widely applied in tissue engineering areas by integrating biomaterials, growth factors, and cells to generate scaffolds for organ regeneration [14,15,16,17].

The design and fabrication of 3D printed scaffolds for the anticipated tissue engineering application requires the development of proper materials and 3D printing methodology. To this end, 3D printing may be achieved by methods such as fused deposition modeling (FDM), stereolithography (SLA), selective laser sintering (SLS), inkjet 3D printing, extrusion-based 3D printing, and binder jet powder bed 3D printing [15,18,19]. Irrespective of the 3D printing method, a typical 3D printing process also includes 3D modeling by using computer-aided design (CAD) software, a 3D scanner, or a photogrammetry procedure. Next, the 3D model is digitized by converting it to an STL file and, subsequently, to a G-code file for 2D layer slicing and, finally, the layer-by-layer printing of the materials.

A wide variety of natural, synthetic, and bioceramic materials have been developed and utilized for 3D bioprinting. Although natural polymers demonstrate ideal biocompatibility, they lack mechanical and electrical properties [20]. Synthetic polymers exhibit poor hydrophilicity, mechanical properties, and cellular compatibility, while bioceramic polymers are prone to cracking and brittleness [21,22]. In addition, material printability, shape retention, gel swelling, and layer-to-layer adhesion may pose additional challenges to engineering 3D printed scaffolds with controlled biological and mechanical properties [23].

To overcome these challenges, nanomaterials, such as carbon nanotubes and graphene, are promising candidates to enhance the strength, stability, and bioactivity of 3D printed constructs [24,25,26]. Among nanomaterials, graphene and graphene-based derivatives have been applied as nanofillers in polymer-based scaffolds. It has been shown that they enhance the mechanical, thermal, electrical, and chemical properties of the scaffolds [27,28,29,30]. Specifically, it has been demonstrated that they improve the strength and toughness of polymer materials by forming a strong bond with their interfaces [31]. Additionally, the tunable chemical surface and the interconnected nano-network structure enhance the extracellular matrix remodeling, regulate cell morphology and adhesion, and promote stem cell differentiation [32,33,34]. Despite that, the dispersity of the graphene sheets, the inadequate bonding with the polymer matrix, and the presence of specific additives may challenge the use of graphene-based material in bioprinting [35,36,37,38,39].

This review discusses the recent advances in graphene and graphene-based derivatives in 3D printing, and their applications in tissue engineering and regenerative medicine. Initially, we will report the utilization of graphene and graphene-based derivatives by different 3D printing approaches. Next, we will report the factors that tune the printability of those materials, as well as the mechanical, chemical, and biological properties of the 3D printed graphene-based constructs. We have summarized the application of those materials for bone, neural, and cardiac tissue regeneration. Finally, we will report the challenges involved in the 3D printing of graphene-based scaffolds for tissue restoration.

## 2. Graphene and Graphene-Based Materials for 3D Printing

**Figure 1:**
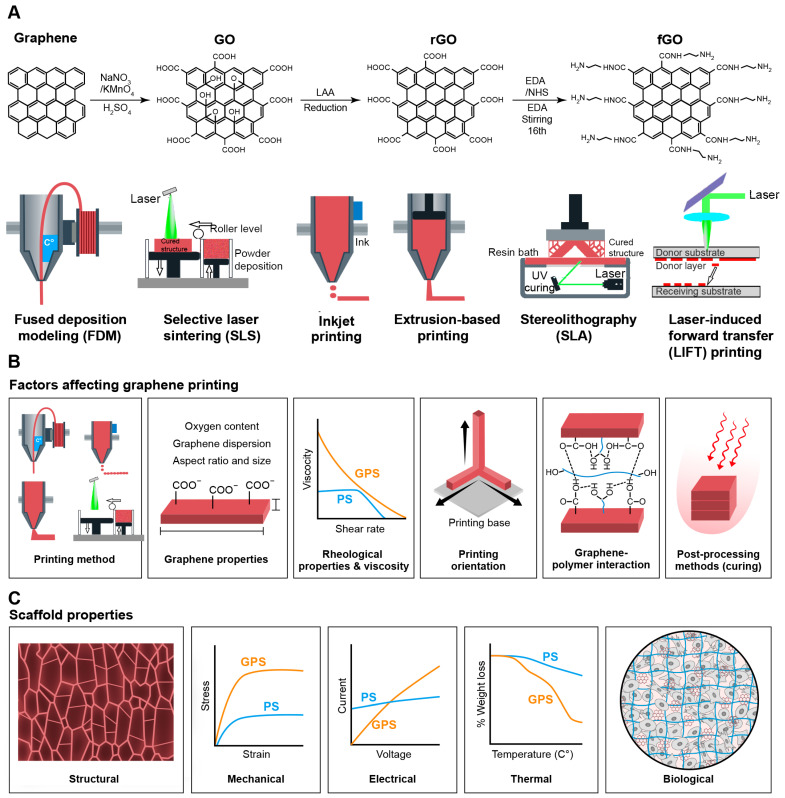
Graphene in 3D printing. (**A**) Graphene-derived materials: graphene, graphene oxide (GO), reduced graphene oxide (rGO), and functionalized graphene oxide (fGO), have been applied to generate 3D printed scaffolds by using different 3D printing approaches, such as fused deposition modeling, selective laser sintering, inkjet printing, extrusion-based printing, stereolithography, and laser-induced forward transfer (LIFT). (**B**) Schematic demonstrates critical factors, including printing method, graphene properties, rheological properties, printing orientation, graphene–polymer interactions, and post-processing methods, which control the 3D printing of graphene-based materials (**C**) Major factors for evaluation of 3D printed graphene-based scaffolds include the characterization of structural, mechanical, electrical, thermal, and biological properties.

Graphene-based materials, such as graphene, graphene oxide (GO), reduced graphene oxide (rGO), and functionalized graphene oxide (fGO), have been synthesized and utilized for 3D printing [40,41] (Figure 1). Table 1 summarizes 3D printing techniques to generate different graphene derivative scaffolds for tissue engineering applications.

### 2.1. Graphene

Graphene is a nanomaterial composed of two-dimensional, hexagonal layers of sp^2^ hybridized carbon atoms forming single-layer, bilayer, and few-layer structures with distinct geometries [42]. The crystalline surfaces are chemically inert, but the edges are active and interact with various chemical groups such as carboxyl (COOH), carbonyl (COH), hydrogenated (CH), and amines (NH_2_) [43,44]. Based on the application, the need to generate single or multi-layer graphene requires different approaches. The most common method involves the control of precise growth kinetics for sheets from small-molecule precursors (bottom-up) or exfoliating bulk graphitic materials (top-down) by using polycyclic aromatic compounds or other molecules with aromatic structures as precursors [45,46,47,48]. An alternate approach involves the dissociation of adjacent graphitic layers in the presence of thermal, compressive, or shear stresses [49]. Finally, carbon nanotubes (CNTs) [50] and fullerenes [51] can also be exfoliated to produce graphene. Irrespective of the method, graphene has unique mechanical, biological, and structural properties, which may be applied in generating 3D tissue constructs [37,52,53]. Specifically, it enhances the scaffold’s mechanical properties and biocompatibility due to its interconnected structure, chemical stability, and surface amenability [54,55,56].

To achieve the fabrication of 3D printed graphene-based scaffolds, various 3D printing approaches have been utilized. Wei et al. were the first to demonstrate the FDM printability of graphene scaffold with polyvinyl alcohol (PVA) and polylactic acid (PLA) [57,58]. Armentia et al. [59] investigated the printability of 0.1 wt% graphene, GO, and graphite nanoplatelets (GOxNP) added to a photopolymerizable acrylic resin by using SLA. The results showed that GO and GOxNP had no effect on printability, but graphene had a negative effect due to the decrease in the polymerization degree. In addition, graphene scaffolds generated by SLA demonstrated limitations in biocompatibility, degradability, and resolution requirements [60]. SLS is an alternative fast and precise 3D printing method with limitations due to the necessity of the presence of high melting temperatures and the employment of post-processing procedures [61,62,63]. Specifically, polyurethane (PU) with 1 wt% graphene nanosheet scaffolds printed by SLS demonstrated 21% and 24% increased in tensile strength and Young’s modulus compared to PU resin, respectively. However, nanosheets with graphene amounts higher than 24 wt% required UV curing. This post-printing process negatively affected the scaffold’s mechanical properties due to interfacial voids and defects on the 3D printed specimens [64]. Also, Chen et al. used SLS to create a 3D printed graphene/polyether ether ketone (PEEK) scaffold with enhanced mechanical properties by incorporating 0.1 and 0.5 wt% graphene [65]. Finally, a 3D inkjet printing approach for engineering graphene/polylactide-co-glycolide (PLG) scaffolds has been developed by Adam et al. Specifically, the 3D printed scaffolds demonstrated high mechanical strength and flexibility, increased electrical conductivity and enhanced proliferation and neuronal differentiation of human mesenchymal stem cells (hMSCs) [66,67].

### 2.2. Graphene Oxide (GO)

GO has been prepared by different approaches, such as the Hummers, the Brodie, and the Staudenmaier methods [68]. The choice of method and parameters significantly impacts the quality and properties of the produced GO [69]. In general, the production of GO requires the oxidization of the graphite powder preparation, followed by exfoliation. Exfoliation methods include sonication, oxidation, or electrochemical reduction. Finally, the resulting GO is purified via dialysis, centrifugation, or filtration [70,71,72].

The use of GO as a material for tissue engineering applications has several advantages. It has been demonstrated to have high mechanical strength and can be easily produced in different forms and sizes, making it suitable for various tissue engineering applications [73,74]. Also, GO has hydrophilic functionalities, such as carboxyl, hydroxyl, and epoxy groups, on its basal plane, making it possible to be functionalized with various biomolecules to promote cell adhesion and differentiation [75,76]. Finally, GO has been shown to be biodegradable, indicating the reduced risk of long-term inflammation or negative consequences of GO for bio-applications [77,78,79].

GO inks are often easier to use in printing the scaffold than graphene inks [80,81]. GO may act as a dispersant, viscosifier, binder, and printing aid for the 3D printing process. Specifically, GO, along with polymers, additives, and solvents, has been developed and utilized as ink for 3D printing applications [82,83]. The utilization of GO in FDM has been challenged due to the high printing temperature, which may trigger oxidation and the loss of electrical properties. Thus, several studies have shown that well-controlled printing parameters such as temperature, extrusion output, printing speed, and printing path were required to eliminate anisotropies and voids in FDM-printed GO scaffolds [58,84,85,86]. Also, GO has been used in SLA printing for various applications, including the generation of microfluidic devices. The presence of GO improved the mechanical and thermal properties of the printed devices, as well as their resistance to UV radiation [87]. Also, a GO–hydroxyapatite (HAP) and polylactic acid (PLLA) biopolymer scaffold have been generated by SLS printing. The presence of GO–HAP in the PLLA/GO–HAP scaffold demonstrated high compressive strength, modulus, and cytocompatibility [88]. Finally, Zhong et al. developed 3D extrusion-based printing with GO/geopolymer (GO/GP) nanocomposites for the first time [19]. In another study, Lee et al. used extrusion-based 3D printing to create GO-reinforced HA/gelatin scaffolds under well-controlled printing conditions [89]. Finally, the 3D printed construct consisting of GO and polyvinyl alcohol (PVA) has demonstrated high conductivity, porosity, and flexibility [90].

### 2.3. Reduced Graphene Oxide (rGO)

rGO is an intermediate structure between graphene and GO that partially restores graphitic characteristics by removing oxygen functional groups through chemical reduction. It restores properties lost during oxidation, recovering 80% of the sp^2^ structure with remaining sp^3^ bonds originating from residual oxygen (C:O = 13:1) [91]. GO is transformed into rGO via reducing agents like hydrazine, sodium borohydride, or hydrogen gas or via chemical, thermal, or electrochemical reduction techniques. Post-reduction of rGO requires purification through dialysis, centrifugation, or filtration. The selection of the method and parameters plays a critical role in determining the quality and properties of the resulting rGO material [92]. It offers improved mechanical strength with a higher tensile strength and Young’s modulus than GO, making it less prone to deformation and capable of supporting greater loads [93]. rGO has also been shown to be biocompatible with a variety of cell types, including hMSCs, making it a promising material for tissue engineering applications [94,95]. To this end, it enhances conductivity in electrospun scaffolds for cardiac and neural tissue restoration.

rGO scaffold has been used in a variety of 3D printing techniques. Sieradzka et al. developed an FDM printing method for fabricating fused filaments from high-impact polystyrene in the presence of rGO [96]. Ajiteru et al. created a printable bioink (SGOB1) for SLA printing by covalently reducing GO with glycidyl methacrylate in silk fibroin. As a result, SGOB1 has been demonstrated as a promising scaffold for brain tissue engineering applications [97]. Yang et al. used SLS to successfully construct a Zn/rGO scaffold. The uniformly dispersed rGO simultaneously increased the strength and ductility of the scaffold while refining the grains and drastically weakening the texture. Moreover, the Zn/rGO scaffold improved cell proliferation and differentiation properties [98]. Finally, extrusion-based 3D printed polycaprolactone (PCL)–rGO composite scaffolds demonstrated that the addition of rGO improved the mechanical properties of printed scaffolds with no adverse effects on the printing process and scaffolds’ biocompatibility [99].

### 2.4. Functionalized Graphene Oxide (fGO)

To generate fGO, the GO surface is modified with a functional group through covalent or noncovalent methods to enhance interactions with polymer and nanoparticles [100]. An alternative process involves the oxidation, carboxylation, nitration, fluorination, and reactivity with a particular reagent, such as porphyrin, for altering the graphene surface to improve biocompatibility, conductivity, and stability [101]. Finally, physical methods use surfactants for functionalization through π-π interactions.

The selection of functional groups and modification methods for the fGO material significantly affected the mechanical and biological properties of the 3D printed scaffolds. For example, graphene has been functionalized with a range of biomolecules, including proteins and nucleic acids [102]. Also, Polyethylene glycol (PEG), a biocompatible polymer, has been widely used for functionalizing graphene [103]. It has been shown that the functionalization of graphene with inorganic nanomaterials, including copper, nickel, alumina, ZnO, iron oxide, etc., imparted electrical, electronic, and magnetic properties to the 3D printed scaffold [104]. Irrespective of the method, fGO has demonstrated high potential to be used for bone, nerve, and muscle tissue engineering applications.

To engineer fGO-based scaffolds, a variety of 3D printing techniques have been developed. Initially, Yang et al. used FDM to show that the covalent polymer functionalized GO/PEEK scaffold had better mechanical and tribological performances than PEEK alone [105]. The 3D printing of the fGO nanocomposite by SLA showed high fidelity, printing repeatability, and mechanical integrity [106]. The addition of SiO_2_ to GO increased the interlayer spacing of GO nanosheets from 0.799 nm to 0.894 nm, resulting in improved dispersion properties. The excellent dispersion of SiO_2_ to GO improved the mechanical characteristics and cytocompatibility of the 3D SLS-printed scaffold [107]. Wajahat et al. described a simple and successful technique for fabricating 2D and 3D micropatterns of Fe_3_O_4_ functionalized graphene-polymer (FGP) nanocomposite employing extrusion-based 3D printing with a highly loaded FGP nanocomposite ink. The ink was stable and suited for 3D printing of FGP items due to the presence of Fe_3_O_4_ nanoparticles (NPs), graphene microflakes (GMFs), and hydroxypropyl cellulose (HPC) [104].

## 3. The Role of Graphene-Based Material Properties in 3D Printing

During 3D printing, graphene’s natural properties, such as strength, conductivity, and surface area, may be compromised, leading to the generation of products with defects. Herein, we describe the parameters that control the 3D printing process and the structural, physical, and chemical properties of the 3D printed scaffolds (Figure 1) [108,109].

### 3.1. Graphene Sheet’s Aspect Ratio

Studies have shown that the aspect ratio controlled the scaffold’s mechanical properties, electrical conductivity, interfacial interactions between polymer/graphene, biocompatibility, and molecule diffusion [110,111,112]. Specifically, a reduced aspect ratio due to sheet agglomeration decreased interfacial interaction, leading to a reduction in the mechanical properties of the scaffold [113]. Although a higher aspect ratio improved mechanical strength, conductivity, and cell attachment, the 3D fabrication process is challenged. Thus, optimization of the aspect ratio is essential for desired 3D printed scaffold properties [114]. Also, graphene enhanced the electrical conductivity of polymers via the conductive network of free electrons that have been formed. Specifically, a study by Nirmalraj et al. showed that the electrical conductivity of graphene nanosheets decreased as the number of layers increased [39]. Finally, Singh et al. found that an increased graphene sheet’s aspect ratio improved the electrical, mechanical properties, and thermal conductivity of acrylonitrile butadiene styrene (ABS) [115].

### 3.2. Graphene-Polymer Interactions

The interfacial interactions of graphene-based nanosheets and polymer controlled the 3D printed scaffolds’ properties. The interaction between the graphene and polymer depends on the sheets’ affinity to polymeric groups and their distribution and alignment along the polymer backbone. The affinity has been mediated through covalent bonding and forces between polar and non-polar polymer chains and oxygen groups of the sheets. GO’s amphiphilic properties advanced the interaction between polar and non-polar polymers [102,103,104]. Hydrophobic polymers favored non-polar covalent interactions with the nanosheets, while polymers with aromatic side groups had π-π interactions with graphene materials functionalized by electron-rich aromatic rings [116,117,118]. Carboxyl and hydroxyl groups on GO interacted with polymer amine groups through hydrogen and epoxy groups on a polymer through covalent bonding, respectively. For example, hydrogen bonding between GO groups and polar polymers (i.e., PEEK/PVA) provided interfacial strength and electrical conductivity. In addition, the presence of van der Waals forces further strengthened this bonding due to the substantial surface area of the nanosheets [63]. Finally, graphene-based materials, such as GO, controlled the binding to the polymers by tuning polymer chain movement and length at different melting temperatures [119]. It has been reported that graphene-based materials enhanced oxidative decomposition and heat adsorption at low loadings (≤1 wt%) and thermal stability at higher loadings (≥5 wt%) [120].

### 3.3. Oxygen Content

Oxygen significantly affected the topography of GO and rGO sheets since GO sheets are hydrophilic and disperse in water, while rGO sheets exhibit hydrophobicity. Specifically, GO reduction in water led to irreversible aggregations, and rGO maintained residual oxygen-containing groups (OCGs) due to limited reduction capabilities. The presence of OCGs allowed the interaction with chitosan, making it water-dispersible through zwitterionic interaction and hydrogen bonding [121]. Although oxidation improved dispersion, it reduced the electrical conductivity of scaffolds for nerve tissue engineering applications [122].

### 3.4. Graphene Dispersion

Since graphene tends to self-aggregate, the dispersion may have reverse outcomes on the 3D printing process and, therefore, on the properties of the scaffold [123]. To achieve a uniform graphene dispersion in a polymer matrix, different methods, such as in situ polymerization, melt compounding, and solvent blending, have been used [124]. However, due to nanosheet dispersion variation, these techniques negatively affected the electrical conductivity of graphene/polyurethane composites [125]. Alternatively, alkaline treatment improved aqueous dispersion, but acidification caused flocculation [126,127]. Thus, it is essential to generate stable aqueous graphene dispersions without compromising graphene’s electrical properties. To this end, electrostatic stabilization is a necessary step for dispersing particles in water, but not in less polar solvents [128,129]. Although the use of surfactants or stabilizing polymers as dispersants improved graphene dispersion, challenges in graphene alignment and 3D printed graphene electronic and structural properties remained [130]. The choice of dispersant altered the graphene sheet’s distribution in the polymer matrix, which affected the UV curing and, overall, the structural integrity of the 3D printed scaffold [131].

### 3.5. Rheological Property and Viscosity

The rheological properties of graphene-based material inks significantly impact the extrusion and flow during the printing. It has been reported that GO suspensions demonstrated shear-thinning behavior, which negatively controlling the extrusion/flow during printing [132]. To overcome those limitations, the presence of surfactants reduces the GO ink viscosity and improves the flow [133]. Also, factors like the selection of polymer and filler nanomaterial concentration are essential to tune ink viscosity. To this end, the presence of polylactic acid (PLA) in graphene-based material ink enhanced the extrusion during printing, but it negatively affected the conductivity of the 3D printed scaffold [66]. Li et al. reported that the rheological properties of GO-containing alginate hydrogel positively correlated with printability. Although alginate/CaCl_2_ hydrogels exhibited low printability due to the thixotropic properties of the ink, the GO addition improved the extrusion and the printing process [134]. Finally, a study on a 3D printed chitosan/GO composite showed that GO improved storage and loss moduli and, ultimately, the overall printing process [135].

### 3.6. Printing Orientation

Different printing orientations (horizontal, oblique, and vertical (0-, 45-, 90-degree)) have affected the properties of the printed resin, as found by Unkovskiy et al. [136]. The presence of magnetic, electric, thermal, and gravitational fields during 3D printing improved the nanomaterial alignment and, therefore, the conductivity and mechanical properties of the scaffold [94]. SLA printing with the partial alignment of graphene–PMMA nanocomposite resins resulted in constructs with enhanced mechanical properties. Printing graphene vertically (perpendicular to the building platform) led to higher tensile strength and modulus than printing it horizontally due to stronger interactions between the layers, as described by Lai et al. [137]. In addition, it has been reported that horizontal layering improved the storage and elastic modulus, and thermal stability due to the alignment of graphene platelets in the direction of tensile loading [86]. Finally, a recent study has demonstrated that printing in the z-axis direction yielded the highest thermal conductivity while printing in the x-y plane had the lowest [138].

### 3.7. Post-Processing Methods

Post-processing is essential to eliminate uncured material by removing supports and washing the 3D printed specimens with ethanol or isopropyl alcohol [139]. However, post-processing approaches resulted in the loss of mechanical and electrical features of the 3D printed scaffolds. In addition, the presence of graphene in scaffolds significantly impacted the photocuring process. Specifically, graphene, as a conductive material, functioned as a photosensitizer, leading to faster and more efficient curing of the scaffolds. However, the specific impact of graphene on the photocuring of scaffolds was dependent on the type and concentration of graphene. Finally, graphene potentially interfered with the curing process by absorbing or scattering light, making the scaffold less transparent and more challenging to cure [140,141,142].

**Table 1:** Methodologies, applications, and challenges of the 3D printed tissue-engineered graphene-based materials.

Material	3D Printing Method	Tissue Engineering Application	Concerns/Challenges	Refs.
**Graphene**				
Graphene/PLA	FDM	Bone, Cardiovascular Neural	large-scale production uniform dispersion of graphene/PLA	[57,58,143]
Graphene/PCL	Extrusion-based 3D printing	Bone	dual functionality: induction of cancer cell death and bone regeneration	[144]
Graphene/PEEK	SLS	Bone	mechanical properties	[63]
Graphene/PLG	Inkjet 3D printing	Bone	ink viscosity surface tension and density large-scale production	[67,145]
Graphene	Extrusion-based 3D printing	Multiple applications	graphene content controlled electrical conductivity of the 3D printed scaffolds	[66]
Graphene/GelMA	Extrusion-based 3D printing	Neural	scalability cost-effectiveness inflammation	[146]
**Graphene oxide (GO)**				
GO	FDM	Multiple applications	high surface energy high printing temperature loss of electrical properties	[58,84,85,86]
GO/HAP/PLLA	SLS	Bone	high GO amount (>12%) loss of mechanical properties	[88]
GO/GP	Extrusion-based 3D printing	Biomedical applications	optimization of 3D printing parameters	[19,89]
GO/fibrin	Extrusion-based 3D printing	Bone	cytotoxicity immunogenicity	[147]
GO/Col aerogel	Extrusion-based 3D printing	Bone	limitations in GO amount (0%, 0.05%, 0.1%, and 0.2% *w*/*v*). mechanical properties biocompatibility	[148]
PCL/GO/Ag/Arg	Extrusion-based 3D printing	Skin	mass ratio pH	[149]
GO/Au/Chitosan	Extrusion-based 3D printing	Cardiovascular	N/A	[150]
**Reduced graphene oxide (rGO)**				
rGO	Extrusion-based 3D printing	Cardiovascular	rGO concentration and dispersion uniform conductivity	[151]
rGO	SLA	Neural	hydrophobicity chemical stability photopolymerization process	[97]
rGO/Zn	SLS	Bone	scalability cost-effectiveness	[98]
rGO/PCL	Extrusion0based 3D printing	Skin	even mixture and distribution of the rGO sheets/PCL matrix	[99]
rGO/ Isabgol	Extrusion-based 3D printing	Skin	uniform dispersion of rGO into isabgol matrix	[152].
rGO/PEA/Chitosan	Extrusion-based 3D printing	Cardiovascular	scalability reproducibility multi-layer tissue constructs	[153]
**Functionalized graphene oxide (fGO)**				
fGO	SLS	Bone	presence of SiO_2_ in situ growth method	[107]
rGO/ Mxene/Hydrogel	Extrusion-based 3D printing	Neural	long-term stability biocompatibility cytotoxicity	[154]
fGO/Fe_3_O_4_/ Polymer	Extrusion-based 3D printing	Biomedical applications	rheological properties nozzle clogging	[104]

3D, three-dimensional; PLA, polylactic acid; PCL, polycaprolactone; PLG, polyactide-co-glycolide; PLLA: poly-L-lactic Acid; GO, graphene oxide; HAP, hydroxyapatite; rGO, reduced graphene oxide; Zn, zinc; PEA, poly ester amide; GelMA: gelatin methacryloyl; fGO, functional graphene oxide; GP, geopolymer; FDM, fused deposition modeling; SLS, selective laser sintering.

## 4. Evaluation of 3D Printed Graphene-Incorporated Polymeric Scaffolds

The printing process and the graphene-based bioinks significantly contribute to the functional properties of the generated 3D products. It is essential to engineer 3D printed constructs with desired properties, which will fit the desired applications. To this end, extensive characterization of their mechanical, thermal, electrical, and biological properties via scanning electron microscope (SEM), atomic force microscopy (AFM), and Kelvin probe force microscopy (KPFM) techniques is necessary [155].

### 4.1. Microstructural and Mechanical Properties Analysis

The 3D printed graphene scaffold’s properties and stability depend on its microstructure, shape, and mechanics. SEM has been employed to examine the internal structure and pore size of 3D printed graphene scaffolds, providing insights into their morphology and architecture. Mechanical properties have been assessed using Instron and dynamic mechanical analysis (DMA) instrumentation. It has been demonstrated that graphene improved the mechanical strength and Young’s modulus of polymers [58]. Studies have shown that the adsorption of polymeric chains on graphene surfaces enhanced their mechanical strength, stiffness, and toughness, making them more suitable for load-bearing applications [33,117,156].

### 4.2. Thermal and Electrical Properties

The graphene–polymer scaffold’s thermal stability and transition temperature have been measured using thermogravimetric (TGA) and differential scanning calorimetric (DSC) calculations. Study by Chen et.al, showed that GO controlled the crystallization and melting temperatures of the neat polymer blend by tuning the chain movement during polymerization polymerization [119]. Graphene enhanced oxidative decomposition and heat adsorption at low loadings (≤1 wt%) and thermal stability at higher loadings (≥5 wt%) in polymer samples [120]. Graphene’s excellent electrical conductivity creates a conductive network for free electrons and improved the electrical conductivity of polymers. Graphene properties like size, surface area, and functionalization generally affected the nanocomposite’s electrical properties. Various techniques, such as the four-point probe method, impedance spectroscopy, conductive atomic force microscopy (C-AFM), and KPFM, have been used to evaluate the electrical conductivity of graphene–polymer scaffolds [38,157]

### 4.3. Biocompatibility

Graphene has tremendous potential in translational medicine, but its biological properties need to be thoroughly investigated. Specifically, the biocompatibility of graphene has been examined through in vitro and in vivo experiments [158]. Graphene’s self-aggregation tendency induced cell damage by disrupting membrane integrity, mediating apoptosis/necrosis, and causing DNA breakage. Additionally, it induced oxidative stress and triggered cytotoxic reactions like lipid peroxidation and DNA damage. Surface modification remains crucial in lowering the immune-inflammatory response and enhancing cell metabolism and proliferation activity [158]. The concentration, size, shape, and surface functionalization of graphene also had a crucial role in mediating cytotoxicity [159]. Some studies have found that by applying chitosan-functionalized GO on the surface of magnesium alloy scaffolds, the corrosion resistance of the scaffolds was increased, and the immune response and growth of vascular endothelial cells were regulated [160]. Finally, healthy volunteers were exposed to 200 gm/m^3^ of graphene oxide nanosheets for 2 h. The study on the first-in-human controlled inhalation of thin GO sheets concluded that the healthy volunteers tolerated it with no adverse effects on cardiorespiratory function, inflammation, or coagulability. This controlled exposure study demonstrated the feasibility of assessing the acute biological effects of graphene oxide in humans, laying the groundwork for further investigations and risk assessments [161]. However, further research is needed to comprehend biocompatibility and metabolic thresholds in clinical settings.

## 5. Applications of 3D Printed Graphene-Based Material in Tissue Engineering

Graphene-based materials have demonstrated the capability to be utilized for tissue engineering and regenerative medicine applications [162,163]. The current section will report their utilization in 3D printed graphene-based scaffolds for soft and hard tissue engineering applications.

### 5.1. Hard Tissue Engineering

Three-dimensional printed graphene-based scaffolds possess mechanical and biological properties to promote bone differentiation and resorption upon tissue regeneration [164]. Xie et al. have demonstrated the osteogenic potential of periodontal ligament stem cells cultured on 3D graphene substrates [165]. Also, 3D printed chitosan/GO scaffolds have been developed and applied for regeneration in critical-size calvarial bone defects [166]. Interestingly, Daneshmandi et.al have developed 3D printed calcium phosphate graphene resorbable and osteoconductive scaffolds [167]. In addition, the presence of GO in 3D printed HAP/gelatine and collagen-based scaffolds reinforced the mechanical properties and bone regeneration potential [89,168]. Also, studies by Li et.al showed that human adipose stem cells differentiated toward osteogenic lineage on 3D printed alginate-based scaffolds coated with GO [82]. Several studies have shown the combination of synthetic polymers, such as PCL, PLLA, PVA, and PLA, and graphene-based materials for the development of 3D printed bone scaffolds with high compressive strength, modulus, cytocompatibility, and bioactivity for osteogenesis [88,169,170,171,172,173]. Finally, 3D printed graphene-based scaffolds have demonstrated tremendous impact on the treatment of osteosarcoma and bone regeneration [144,174]. Overall, these studies have demonstrated the direct application of 3D printed graphene-derived materials tissue scaffolds for bone regeneration and restoration.

### 5.2. Soft Tissue Engineering

**Figure 2:**
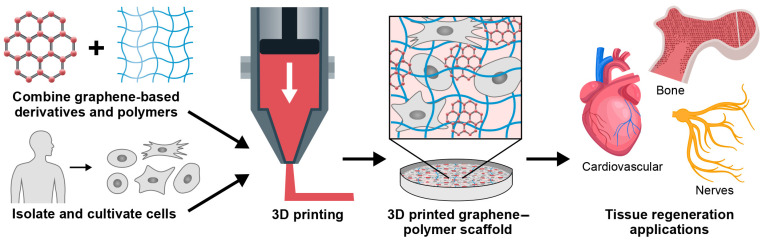
Schematic of generating 3D printed tissue engineered graphene-based scaffolds. Graphene-derived materials combined with cells are 3D printed to generate scaffolds for bone, neural, and cardiac tissue regeneration.

Although successful hard tissue regeneration requires enhanced mechanical properties of the 3D graphene-based scaffold, its electrical conductivity and structural properties make it unique for soft tissue engineering applications. Herein, we will focus on the application of the 3D printed scaffolds for neural and cardiac regeneration (Figure 2).

#### 5.2.1. Nerve Tissue Engineering

Studies have shown that graphene combined with anticoagulants and growth factors induced nerve cell growth and differentiation and, overall, promoted nerve regeneration [175,176]. Specifically, it has been shown that graphene enhanced adhesion, differentiation, and the bioelectricity of hNSCs [177]. Although many scaffolds have been generated for neural tissue engineering applications, herein, we will focus on the generation and application of the 3D printed scaffold in regenerative medicine. Vijayavenkataraman et al. utilized a 3D PCL–rGO bioprinted scaffold for repairing peripheral nerve injuries. The presence of rGO in those scaffolds increased neural cell proliferation differentiation of P12 cells [178]. Qian et al. used the layer-by-layer casting (LBLC) method to produce 3D printed graphene-based scaffolds for neural tissue engineering applications [179]. Huang et al. [146] developed a 3D graphene mesh tube filled with alginate and GelMA double network hydrogel, which has appropriate mechanical strength and conductivity for supporting the proliferation and arrangement of RSC96 nerve cells. The GMT/DN hydrogel scaffold, with a Netrin-1 concentration of 100 mg/mL, promoted Schwann cell migration and the endothelial tubular shape, leading to effective peripheral nerve regeneration. However, complications such as inflammation, oxidative stress, fibrosis, and inadequate vascular formation hindered the success of regeneration. Adam et al. developed 3D printed graphene/PLG scaffolds and demonstrated enhanced proliferation and neuronal differentiation of hMSCs [66,67]. Ajiteru et al. created a printable bioink (SGOB1) for SLA printing by covalently reducing GO with glycidyl methacrylate in silk fibroin. As a result, SGOB1 has been demonstrated as a promising scaffold for brain tissue engineering applications [97]. Finally, the studies suggested that incorporating GO and rGO in silk-based scaffolds enhanced the metabolic activity, proliferation, and neurite extension of neuronal cells, making them promising substrates for neural tissue engineering applications [180].

#### 5.2.2. Cardiovascular Tissue Engineering

Graphene in 3D cardiac scaffolds enhances electroconductivity, anisotropic nano topology, and improves mechanical properties. Shin et al. [153] developed a 3D cardiac tissue assembly method using a layer-by-layer approach with GO-based thin films as an adhesive layer. This method improved the maturation and electrical coupling of cardiac cells by incorporating three different cell types (hMSCs, cardiomyocytes, and endothelial cells). Saravanan et al. found that the implantation of a GO–Au nanosheet containing a chitosan scaffold improved ventricular contractility and function in the infarcted heart [150]. The GO-based film also improved the survival rate of hMSCs in vivo by acting as a shield against reactive oxygen species. Despite that, the optimization of GO concentration for cardiac tissue engineering that balanced conductivity and scaffold porosity, was essential [151,181]. Karimi et al. found that the combination of alginate and rGO showed improved biocompatibility for cardiac repair [182]. The incorporation of rGO into gelatin methacryloyl (GelMA) hydrogels improved their electrical conductivity and mechanical properties, making them suitable for cardiac tissue engineering [94]. Overall, graphene-based polymeric scaffolds promote cardiac regeneration by improving the function and healing of the scar area.

## 6. Challenges and Future Directions in the 3D Printing of Graphene-Based Scaffolds

Many studies have reported the potential broad application of graphite in biomedical areas due to its low cost and abundance in nature. Despite this, the high-scale production of graphene-derived materials, such as GO, from graphite is costly and laborious [183,184]. These limitations may negatively impact the utilization of graphene-derived materials for 3D bioprinting applications due to the large amount of the material and the advanced equipment that is required.

Although different fabrication methods have been utilized to generate 3D graphene-based scaffolds, the utilization of a 3D bioprinting approach for engineering 3D printed graphene/polymer tissue-engineered scaffolds demonstrate limitations and challenges (Table 1). Studies have shown that each 3D printing approach may present challenges to generating 3D graphene-based tissue engineered scaffolds due to the utilization of high temperature, low resolution and scalability, and high cytotoxicity. For example, scaffolds generated via SLS and SLA printing approaches have shown low cost-effectiveness, mechanical properties, and scalability. Also, those methods require the application of post-printing processes, which may negatively affect the scaffold functionality [60,61,62,63]. FDM bioprinting faces challenges in achieving high resolution and adequate cell viability due to the use of temperature-sensitive bioinks [90]. Extrusion-based techniques deposit the inks layer by layer, providing material versatility, while the generated 3D printed scaffolds often demonstrate low resolution, biocompatibility, and mechanical properties. Future directions for the generation of 3D printed graphene-based scaffolds, to avoid the limitations of the current methods, may involve the use of the laser-induced forward transfer (LIFT) method [185,186,187].

Additional challenges to successfully generating 3D printed scaffolds may be raised because of the lack of proper criteria and measurements for the bioinks and the final 3D printed products. The characterization of the rheological properties of the graphene-based ink, aspect ratio dispersion, polymer/graphene ratio, and oxygen content may be essential parameters to achieve 3D printed scaffolds with controlled mechanical, electrical, and biocompatible properties. Finally, the high cytotoxicity of graphene may raise significant challenges in the translational application of the 3D printed graphene-based constructs for bone, neural, and cardiovascular regeneration applications. Interestingly, the metabolic fate of graphene in vivo has not been fully explored, raising concerns regarding the negative impact of prolonged graphene use on the body. Thus, further research needs to be conducted to unravel how graphene is metabolized in the body. Finally, those limitations dictate the need to develop new bio-graphene inks [188,189], which may be utilized for generating 3D printed tissue engineered scaffolds with advanced regenerative and restoration capacity [190].

## 7. Conclusions

This review focuses on 3D printing of graphene scaffolds for tissue engineering applications. Graphene has a distinctive 2D structure with exceptional mechanical and electrical properties, versatile surface chemistry, rough morphology, and impermeability. These properties are essential for generating tissue engineered scaffolds. We reviewed various graphene derivative scaffolds created by different 3D printing techniques. Moreover, we reported different factors influencing the printing process and the mechanical, chemical, and biological properties of the 3D printed scaffolds. Finally, we showed the utilization of graphene in bone, neural, and cardiac tissue regeneration. We described the underlying mechanisms and distinctive properties of graphene-based materials for each application. For instance, electric conductivity is necessary for cardiac and nerve regeneration, while mechanical strength is necessary for bone and cartilage regeneration. It is important to note that in most cases, the functions of those materials in tissue engineering applications are attributed to the synergistic effect of two or more properties. Despite graphene’s challenges, such as toxicity and side effects, utilization of graphene in 3D bioprinting may pave the way to generate new regenerative-based strategies with potential translational implications in orthopedics, neurology, and cardiovascular areas.

## Abbreviations

3D	Three-dimensional
CAD	Computer-aided design
FDM	Fused deposition modeling
SLA	Stereolithography
SLS	Selective laser sintering
GO	Graphene oxide
rGO	Reduced graphene oxide
fGO	Functionalized graphene oxide
CNTs	Carbon nanotubes
GOxNP	Graphite nanoplatelets
PVA	Polyvinyl alcohol
PLA	Polylactic acid
PLLA	Poly-L-lactic acid
PU	Polyurethane
PLG	Polylactide-co-glycolide
PCL	Polycaprolactone
HAP	Hydroxyapatite
PEA	Poly ester amide
hMSCs	Human mesenchymal stem cells
GP	Geopolymer
PEEK	Polyether ether ketone
PEG	Polyethylene glycol
FGP	Fe_3_O_4_ functionalized graphene polymer
GMFs	Graphene microflakes
HPC	Hydroxypropyl cellulose
NPs	Nanoparticles
ABS	Acrylonitrile butadiene styrene
OCGs	Oxygen-containing groups
hNSCs	Human neural stem cells
LBLC	Layer-by-layer casting
GelMA	Gelatin methacryloyl
LIFT	Laser-induced forward transfer.
SEM	Scanning electron microscope
AFM	Atomic force microscopy
KPFM	Kelvin probe force microscopy
TGA	Thermogravimetric
DSC	Differential scanning calorimetric
DMA	Dynamic mechanical analysis
